# Evaluation of CDC’s Hemophilia Surveillance Program — Universal Data Collection (1998–2011) and Community Counts (2011–2019), United States 

**DOI:** 10.15585/mmwr.ss6905a1

**Published:** 2020-09-04

**Authors:** Laura A. Schieve, Vanessa R. Byams, Brandi Dupervil, Meredith A. Oakley, Connie H. Miller, J. Michael Soucie, Karon Abe, Christopher J. Bean, W. Craig Hooper

**Affiliations:** 1 Division of Blood Disorders, National Center on Birth Defects and Developmental Disabilities, CDC

## Abstract

**Problem/Condition:**

Hemophilia is an X-linked genetic disorder that primarily affects males and results in deficiencies in blood-clotting proteins. Hemophilia A is a deficiency in factor VIII, and hemophilia B is a deficiency in factor IX. Approximately one in 5,000 males are born with hemophilia, and hemophilia A is about four times as common as hemophilia B. Both disorders are characterized by spontaneous internal bleeding and excessive bleeding after injuries or surgery. Hemophilia can lead to repeated bleeding into the joints and associated chronic joint disease, neurologic damage, damage to other organ systems, and death. Although no precise national U.S. prevalence estimates for hemophilia exist because of the difficulty identifying cases among persons who receive care from various types of health care providers, two previous state-based studies estimated hemophilia prevalence at 13.4 and 19.4 per 100,000 males. In addition, these studies showed that 67% and 82% of persons with hemophilia received care in a federally funded hemophilia treatment center (HTC), and 86% and 94% of those with the most severe cases of hemophilia (i.e., those with the lowest levels of clotting factor activity in the circulating blood) received care in a federally funded HTC. As of January 2020, the United States had 144 HTCs.

**Period Covered:**

1998–2019.

**Description of the System:**

Surveillance for hemophilia, which is a complex, chronic condition, is challenging because of its low prevalence, the difficulty in ascertaining cases uniformly, and the challenges in routinely characterizing and tracking associated health complications. Over time, two systems involving many stakeholders have been used to conduct ongoing hemophilia surveillance. During 1998–2011, CDC and the HTCs collaborated to establish the Universal Data Collection (UDC) surveillance system. The purposes of the UDC surveillance system were to monitor human immunodeficiency virus (HIV) and bloodborne viral hepatitis in persons with hemophilia, thereby tracking blood safety, and to track the prevalence of and trends in complications associated with hemophilia. HTC staff collected clinical data and blood specimens from UDC participants and submitted them to CDC. CDC tested specimens for viral hepatitis and HIV. In 2011, the UDC surveillance system was replaced by a new hemophilia surveillance system called Community Counts. CDC and the HTCs established Community Counts to expand laboratory testing and the collection of clinical data to better identify and track emerging health issues in persons with hemophilia.

**Results:**

This report is the first comprehensive summary of CDC’s hemophilia surveillance program, which comprises both UDC and Community Counts. Data generated from these surveillance systems have been used in the development of public health and clinical guidelines and practices to improve the safety of U.S. blood products and either prevent hemophilia-related complications or identify complications early. Several factors have played a role in the effectiveness of the UDC and Community Counts systems, including 1) a stable data collection design that was developed and is continually reviewed in close partnership with HTC regional leaders and providers to ensure surveillance activities are focused on maximizing the scientific and clinical impact; 2) flexibility to respond to emerging health priorities through periodic updates to data collection elements and special studies; 3) high data quality for many clinical indicators and state-of-the-art laboratory testing methods for hemophilia treatment product inhibitors (developed and refined in part based on CDC research); 4) timely data and specimen collection and submission, laboratory specimen testing, analysis, and reporting; and 5) the largest and most representative sample of persons with hemophilia in the United States and one of the largest and most comprehensive data collection systems on hemophilia worldwide.

**Interpretation:**

CDC has successfully developed, implemented, and maintained a surveillance system for hemophilia. The program can serve as an example of how to conduct surveillance for a complex chronic disease by involving stakeholders, improving and building new infrastructure, expanding data collection (e.g., new diagnostic assays), providing testing guidance, establishing a registry with specimen collection, and integrating laboratory findings in clinical practice for the individual patient.

**Public Health Action:**

Hemophilia is associated with substantial lifelong morbidity, excess premature deaths, and extensive health care needs throughout life. Through monitoring data from Community Counts, CDC will continue to characterize the benefits and adverse events associated with existing or new hemophilia treatment products, thereby contributing to maximizing the health and longevity of persons with hemophilia.

## Background

### Overview of Hemophilia 

Hemophilia refers to a group of genetic disorders resulting in deficiencies in blood-clotting proteins. Hemophilia A (also known as classic hemophilia) is a deficiency of factor VIII, and hemophilia B (also known as Christmas disease) is a deficiency of factor IX (*1*,*2*). Because both hemophilia A and B are X-linked disorders, they primarily affect males. Although no precise national U.S. prevalence estimates exist for hemophilia because of the difficulty identifying patients who receive care from various types of health care providers, two in-depth, state-based studies (one of persons in six states in 1995 and one of persons in one state during 2011–2013) estimated hemophilia prevalence as 13.4 and 19.4 per 100,000 males, respectively (*3*,*4*). Approximately one in 5,000 males are born with hemophilia, with hemophilia A about four times as common as hemophilia B (*3*). Both disorders are characterized by spontaneous internal bleeding and excessive bleeding after injuries or surgery. Hemophilia can lead to repeated bleeding into the joints, which results in chronic joint disease, pain, and mobility limitations. Hemophilia bleeding also might cause neurologic damage, damage to other organ systems, and death. The extent of joint disease and other complications varies by the severity of the hemophilia (*5*), which is defined based on the level of factor activity in the circulating blood. Thousands of different variants of the respective genes coding for factor VIII and factor IX have been identified to cause hemophilia, leading to variability in both the severity and bleeding phenotypes observed for both hemophilia A and B (*1*,*2*).

As early as the second century AD, references were made to a bleeding disorder that clustered in males within certain families (*6*). Case series of this condition were reported over the centuries, and the term hemophilia, meaning “affinity to blood,” was first used to describe the condition in 1838. In the 1800s, scientists and clinicians recognized that hemophilia involved a deficit in the body’s coagulation processes and was linked to genetics. However, the underlying pathophysiology of factor VIII and factor IX deficiencies that cause hemophilia was not understood until the 1940s and 1950s. In the 1950s and 1960s, treatment for hemophilia consisted primarily of whole blood or fresh plasma transfusions, both of which had inadequate levels of clotting factors to control severe bleeding, such as that associated with surgery or trauma. 

Consequently, before the 1970s, life expectancy estimates in various developed countries for persons with severe hemophilia were in the teens or early 20s (*7*). In the mid-1960s, factor VIII concentrates derived from plasma were developed, and in the 1970s, lyophilized factor VIII concentrate treatments revolutionized hemophilia care, resulting in both extended life expectancies and vast improvements in quality of life (*6*). However, the gains in life expectancy for hemophilia patients were short lived. Factor concentrates, which were prepared from large pools of human plasma, were contaminated and associated with high risk for bloodborne infections, most notably human immunodeficiency virus (HIV), which infected an estimated 60%–70% of persons with severe hemophilia by the early 1980s, and hepatitis C virus (HCV), which infected nearly all persons with severe hemophilia (*8*). By 2002, HIV infection and acquired immune deficiency syndrome (AIDS) resulted in the deaths of nearly 40% of the estimated 10,000 persons with hemophilia living in the United States in 1977 (*9*).

The incidence of HIV and HCV infection among persons with hemophilia decreased during the 1990s because of safer blood products (e.g., heat inactivation of HIV present in treatment products derived from human plasma) and the development of recombinant (non–blood-based) factor VIII and factor IX products (*9*,*10*). Hemophilia patient advocates, providers, and CDC established surveillance systems to track the safety and relative efficacy of hemophilia treatment approaches and to better understand the health care needs of persons with hemophilia.

### History of CDC Hemophilia Surveillance Activities 

In 1975, the Health Resources and Services Administration (HRSA) received a congressional appropriation of funding to develop a program to support an integrated regional network of hemophilia treatment centers (HTCs) (Pub L. 94–62), the precursor to CDC’s current hemophilia surveillance activities (Box 1). The foundation of HTC care is the comprehensive care model, defined as the delivery of integrated, multidisciplinary clinical care to persons with hemophilia and other bleeding disorders and their families; comprehensive care and services include access to subspecialists, specialized laboratory diagnostics, treatment programs, patient education, and support services (*11*–*13*). HRSA grant funding continues to support the comprehensive care model at HTCs. In addition, the HTC network became integral to establishing CDC’s future public health surveillance system. In 1983, Congress appropriated funding for CDC to provide AIDS risk reduction services for persons with hemophilia and for others who used blood-based treatment products. (Because the causative pathogen for AIDS was not characterized until 1984, the funding appropriated in 1983 specified risk reduction services for AIDS rather than HIV.) CDC established partnerships with HTCs to develop and implement strategies to prevent AIDS in persons with hemophilia. In 1989, CDC, HRSA, and the HTCs developed the hemophilia minimal dataset to track HIV risk reduction services at HTCs. In the 1990s, CDC and the HTCs expanded their activities in response to congressional funding to develop a public health program to reduce bleeding disorder complications. In 1995, CDC, in collaboration with the health departments in six states (Colorado, Georgia, Louisiana, Massachusetts, New York, and Oklahoma), established the Hemophilia Surveillance System, which was used to conduct active, population-based surveillance to understand the prevalence of hemophilia and its associated illnesses, complications, and deaths. Data were collected during 1995–1999 for persons receiving care both within and outside of HTCs. Among other findings, the data indicated that approximately two thirds of persons with hemophilia who were living in these states were receiving care in an HTC, with an even higher proportion (86%) of those with severe hemophilia receiving care in an HTC (*3*). In addition, care in an HTC was associated with increased survival (*14*). A more recent study using the same population-based methodology in one state estimates that 82% of persons with hemophilia and 94% of those with severe hemophilia receive care in a federally funded HTC (*4*).

BOX 1 **History of CDC hemophilia surveillance activities**

- **1975:** Health Resources and Services Administration (HRSA) receives congressional appropriation of funding to establish a program to support comprehensive care for persons with hemophilia and their families through a network of regional centers called hemophilia treatment centers (HTCs).
- **1983:** CDC receives congressional appropriation of funding to work with HTCs to reduce the risk for acquired immunodeficiency syndrome* among persons with hemophilia and others who used blood product treatments.
- **1989:** CDC, HRSA, and HTCs collaborate on the development of the hemophilia minimal data set to track human immunodeficiency virus risk reduction services at HTCs.
- **1995:** CDC establishes the Hemophilia Surveillance System to conduct population-based surveillance of hemophilia prevalence in six states (Colorado, Georgia, Louisiana, Massachusetts, New York, and Oklahoma).
- **1998:** CDC, in partnership with HTCs, establishes the Universal Data Collection (UDC) surveillance system, a national, HTC-based system for longitudinal tracking of bleeding disorder complications, with a focus on bloodborne infections and joint disease.
- **2010:** CDC convenes a stakeholder meeting to review the UDC system, with a focus on possible updates to collect data on emerging health issues.
- **2011:** CDC, in partnership with HTCs, establishes Community Counts, the next (expanded) iteration of the HTC-based surveillance system for persons with bleeding disorders who receive care from HTCs. Because Community Counts built on UDC’s data collection method for many indices, the data from both systems can be combined to assess long-term trends.

* Because the causative pathogen for AIDS was not characterized until 1984, the funding appropriated in 1983 specified risk reduction services for AIDS rather than human immunodeficiency virus.

In 1998, CDC, in collaboration with the HTCs, established the Universal Data Collection (UDC) surveillance system to monitor HIV and bloodborne viral hepatitis in persons with hemophilia, thereby tracking blood safety, and to track the prevalence of and trends in complications associated with hemophilia with a focus on infectious diseases and joint disease. In 2010, CDC convened a meeting with key stakeholders in the blood disorders community, including representatives from HTCs and blood banks, professional medical organizations, research organizations, consumer organizations, and other federal health agencies, to obtain clinical perspectives on emerging health needs in the bleeding disorders population. Information from this stakeholder discussion was used to develop the next iteration of the surveillance system. This system, known as the Community Counts Public Health Surveillance of Bleeding Disorders project (Community Counts), replaced UDC. Community Counts builds on UDC but has expanded data collection in several ways. In addition to tracking bloodborne infections and joint disease complications, Community Counts includes more in-depth tracking of other hemophilia complications and hemophilia treatment complications (e.g., antibodies [inhibitors] to treatment products). Community Counts also collects additional data on health indices (e.g., cancer and cardiovascular disease) with special consideration of health conditions relevant to the growing aging hemophilia population.

## Comparison of UDC (1998–September 2011) and Community Counts (October 2011–Present)

Both UDC and Community Counts collect data on persons with bleeding disorders other than hemophilia such as von Willebrand disease. Because hemophilia was the impetus for the development of the surveillance program, this report exclusively describes the evaluation of UDC and Community Counts surveillance systems in tracking the hemophilia population. Neither UDC nor Community Counts differentially classifies males and females with factor VIII or IX deficiency. Both are reported as cases of hemophilia. Although carrier status for X-linked genetic conditions such as hemophilia has historically been considered benign, women with a genotype conventionally considered indicative of hemophilia carrier status have been shown to exhibit extensive heterogeneity in both level of circulating clotting factor activity and risk for bleeding events. Certain women produce very little clotting factor because of the X chromosome inactivation pattern established during the embryonic period. Moreover, even women who have only mildly low levels of circulating clotting factor have been found to have increased risk for bleeding in joints or after tooth extraction or surgery, and anemia attributed to menstrual blood loss than women who are not carriers (*15*).

Although Community Counts was designed to align with UDC so that data from the two systems could be combined to assess long-term trends, the systems have several differences (Table 1). In general, Community Counts collects more data than UDC, providing a more comprehensive assessment of the health and health care needs of hemophilia patients.

**TABLE 1:** Comparison of the Universal Data Collection and the Community Counts surveillance systems

System details	Universal Data Collection system	Community Counts system
Time frame	1998 through September 2011	October 2011 to present (however, the full data collection system was not implemented until 2013)
Primary focus	Track blood safety through HIV and viral hepatitis monitoring Track hemophilia complications with a focus on infections and joint disease	Continued tracking of bloodborne infections and joint disease Additional, more in-depth tracking of other hemophilia complications and additional indices relevant to the aging hemophilia population
Data source	Federally funded HTCs	Federally funded HTCs
Funding	Cooperative agreement awarded to 12 HTC regional centers	Cooperative agreement awarded to ATHN to serve as Community Counts coordinating center; ATHN administers subcontracts with HTC regional centers, which then administer contracts to individual HTCs
Partnerships	USHTCN: includes regional medical directors, regional coordinators and health care providers at individual HTCs Multidisciplinary committees with representation from CDC and USHTCN established to advise on all aspects of UDC implementation	USHTCN ATHN Multidisciplinary committees with representation from CDC, USHTCN, and ATHN established to advise on all aspects of Community Counts implementation
System components	Patient registry: clinical data from medical records and direct patient inquiry Laboratory specimens Mortality reporting	Patient registry: clinical data from medical records and direct patient inquiry Laboratory specimens Mortality reporting HTC population profile: minimal data collection about the entire hemophilia population served by HTCs
Consent for registry participation	UDC designated by CDC as research Participants (or parents of minor children) asked to provide informed consent	Community Counts designated by CDC as nonresearch public health surveillance not requiring consent Participants still asked to provide written authorization for participation in the registry
Patient registry clinical data forms	Initial visit form: historic and current clinical data Subsequent annual visit forms: clinical information since the last registry submission	Initial visit form: historic and current clinical data Subsequent annual visit forms: clinical information since the last registry submission
Patient registry: types of data reported on clinical forms	Demographics Weight and height Family history History of HIV, hepatitis C, and liver disease Bleeding disorder diagnoses Treatment regimen and products Bleeding episodes Mobility restrictions and joint procedures HTC laboratory results, including levels of circulating clotting factor and inhibitor titers Joint range of motion HIV risk reduction measures Optional supplemental quality of life questionnaire (since 2005)	Demographics Weight and height Family history History of HIV, hepatitis C, and liver disease Bleeding disorder diagnoses Treatment regimen and products Bleeding episodes: more extensive info than UDC Mobility restrictions and joint procedures HTC laboratory results, including levels of circulating clotting factor and inhibitor titers Additional information on patient inhibitors to treatment products Chronic pain Opioid use for chronic pain Health care use Comorbid medical conditions (e.g., cancer and cardiovascular disease)
Patient registry data submission	Primary submission source throughout UDC: paper forms submitted through U.S. postal system, although some HTCs developed and submitted electronic forms via secure FTP Forms entered and transferred to electronic database and reviewed; HTCs asked to resolve data discrepancies and provide missing data HTCs encouraged to submit data continuously rather than submitting in batches	Before 2015: paper forms submitted through U.S. postal system and entered into an electronic database Since 2015: data submitted electronically via an online data capture system developed and maintained by ATHN Review of forms submitted on paper and electronically; HTCs asked to resolve data discrepancies and provide missing data Some data checks integrated into ATHN system (i.e., to occur in real time) HTCs encouraged to submit data continuously rather than in batches
Laboratory specimens and tests	Serum specimens Hepatitis A, B, and C HIV Plasma specimens (for select years) Hepatitis C RNA	Serum specimens Hepatitis C HIV Plasma specimens Inhibitors to treatment products
Laboratory specimen shipping timeline	Centrifuged and shipped to CDC on cold packs within 30 hours of blood draw	Centrifuged and shipped to CDC on cold packs within 72 hours of blood draw or frozen and shipped on dry ice within 30 days of blood draw
Laboratory accreditation	CLIA-certified laboratory Tests use FDA-approved kits or CLIA-approved in-house developed tests	CLIA-certified laboratory Tests use FDA-approved kits or CLIA-approved in-house developed tests
Biobank	Both serum and plasma specimens stored long term (with participant permission)	Both serum and plasma specimens stored long term (with participant permission)
Publication of surveillance data	Publication of key findings in *MMWR* or peer-reviewed journals Periodic comprehensive surveillance reports published on CDC website	Publication of key findings in *MMWR* or peer-reviewed journals Periodic comprehensive surveillance reports published on CDC website Data visualization tool

**Abbreviations:** ATHN = American Thrombosis and Hemostasis Network; CLIA = Clinical Laboratory Improvement Amendments; FDA = U.S. Food and Drug Administration; FTP = file transfer protocol; HIV = human immunodeficiency virus; HTC = hemophilia treatment center; UDC = Universal Data Collection; USHTCN = U.S. HTC Network.

### Data Source, Funding, and Core Partnerships

Persons are enrolled in UDC and Community Counts through HTCs, which collect and submit data and specimens to CDC. Thus, the U.S. HTC Network (USHTCN), which includes the regional medical directors, regional coordinators, and the health care providers, project coordinators, and data managers at the federally funded HTCs, was and remains a critical partner in the development, implementation, and maintenance of both UDC and Community Counts. As of January 2020, the United States has 144 HTCs.

UDC was funded through cooperative agreements in 1996, 2001, and 2006; each funding cycle included awards to the 12 HTC regional centers in USHTCN at that time. Community Counts is funded through a cooperative agreement awarded to the American Thrombosis and Hemostasis Network (ATHN), a nonprofit organization that serves as the Community Counts coordinating center. ATHN works in partnership with USHTCN and administers subcontracts with HTC regional core centers that then administer contracts to HTCs in their region (*16*). The USHTCN collaborators provide clinical expertise related to hemophilia care and complications as well as administrative insights on how to best reach patients and execute the project. ATHN promotes technology tools to advance care and research for persons with bleeding disorders and provides the infrastructure and platform for HTCs to electronically record and transmit their data to Community Counts.

Similar to UDC’s infrastructure, the infrastructure of Community Counts includes several multidisciplinary committees with representation from CDC and USHTCN and now also includes ATHN representation. These committees discuss and advise on the overall direction of the project and the analytic priorities, support the administration of Community Counts in each of the regions, and review analysis proposals and manuscripts and other data reports.

### Surveillance System Components

The UDC system included two components: 1) a patient registry, in which HTCs actively collected and submitted data and specimens on their current hemophilia patients and 2) a mortality reporting component, in which HTCs submitted data on the characteristics of decedents and causes of death. The patient registry included two subcomponents: 1) submission of clinical data collected through abstraction of patients’ medical records and direct patient inquiry and 2) submission of laboratory specimens.

Community Counts includes the two UDC components (a patient registry component with clinical data and laboratory specimen submissions and a mortality reporting component) and a new third component, the HTC population profile, which collects basic information on the entire hemophilia population who received services at an HTC in a given year. Patients who agree to participate in the patient registry are a subset of patients included in the HTC population profile. This addition to the surveillance system allows for assessment of the proportion and characteristics of the population participating in the registry compared with the entire hemophilia patient population served by HTCs.

Both UDC and Community Counts collect registry data and specimens at the patient’s initial visit and thereafter at subsequent annual visits. Typically, data are collected at the patient’s annual HTC comprehensive care visit. However, data might not be available annually for each patient because the frequency of HTC patient visits is related to diagnosis, severity, and complications.

When UDC was established, CDC classified the data collection as a research project because available data were sparse on certain complications and, in addition to health monitoring, UDC data analyses were expected to make important, generalizable contributions to advancing the understanding of hemophilia. Thus, UDC received CDC institutional review board approval, and participants (or parents/guardians of minor children) were asked to consent to the sharing of their information with CDC. Because the primary focus of Community Counts, which was established more than a decade after UDC, was continued public health monitoring of hemophilia complications, the program was designated as nonresearch public health surveillance, which does not require consent; regardless, the program still requires that participants provide written authorization to participate in the registry.

CDC collected data through the UDC system from 1998 through September 2011; during this time, patient registry data were collected on approximately 77,400 visits from approximately 18,800 persons with hemophilia. Community Counts was established in October 2011. Data submission for the Community Counts HTC population profile submission began in 2012, although submissions to the patient registry and mortality reporting system were both initiated in 2013. As of January 2020, Community Counts patient registry data have been collected on approximately 31,000 visits from approximately 12,000 persons with hemophilia.

#### Patient Registry: Clinical Form Data Collection and Submission

HTC staff use standardized forms to collect clinical data for UDC and Community Counts. An initial clinic form that includes historic and current (at the time of visit) clinical information is collected once for each patient. Thereafter, at the annual patient visits, a shorter form is used to collect clinical information since the last registry submission. Thus, both UDC and Community Counts include a longitudinal component whereby an individual’s progression on health indices can be tracked and variations in individual health progressions can be examined across the hemophilia population. Because annual visits might not occur exactly at 1-year intervals, the minimum time required between annual visit submissions is 9 months. 

Community Counts and UDC both collect data on demographics; weight and height to calculate body mass index; family history of bleeding disorders; patient history of HIV, HCV, and liver disease; bleeding disorder diagnoses; treatment regimen and products used; bleeding episodes; and previous HTC laboratory results pertaining to clotting factor levels and inhibitor detection. Additional data collected by Community Counts include more extensive information on inhibitor and bleeding episodes, chronic pain, opioid use for chronic pain, health care use, and comorbid medical conditions. In addition, collection of certain UDC data was discontinued in Community Counts, including data on joint range of motion measurements, HIV risk reduction information, and an optional supplemental quality of life questionnaire.

UDC and Community Counts patient registry forms submitted to CDC are deidentified, and patients are assigned unique identification numbers generated by software programs. Only HTCs are able to link the data to patient personal identifiers. However, for both UDC and Community Counts, the software programs used to generate identification numbers have features to deduplicate records within and across HTCs. Therefore, if a person receives care at more than one HTC, the same identification number will be provided at each HTC (although none of the identifying information used in the deduplication procedure is transmitted across HTCs or to CDC). Before 2015, the primary method of data submission was on paper through the U.S. postal system. In the later years of UDC, some HTCs developed and used an electronic version of the forms, which they submitted via secure FTP (file transfer protocol). Submitted data were entered (or transferred if submitted electronically) into an electronic database and reviewed, and HTCs were asked to resolve discrepancies and provide missing data. Since 2015, data have been submitted electronically through an online system created and maintained by ATHN. Using the unique system-generated identifiers, the surveillance forms are automatically populated with certain data already existing in the electronic database, and other data are directly entered into the system. Certain data field and form validations are integrated into the system to identify potential discrepancies and incomplete data. In addition, several features of the ATHN system have made the data collection easier; for example, the system includes an up-to-date list (updated daily) of treatment products approved by the U.S. Food and Drug Administration (FDA). For both paper and electronic data submissions, HTCs have always been encouraged to submit data to CDC continuously (at the time of collection), rather than submitting forms in batches.

#### Patient Registry: Laboratory Specimen Collection, Testing, and Results Reporting

In addition to collecting laboratory specimens for clinical testing as part of comprehensive care, HTCs also obtain blood specimens from UDC and Community Counts participants during each visit included in the registry and submit them to CDC. These specimens are limited to certain tests deemed to be important to CDC’s surveillance efforts. The testing conducted on the specimens has evolved over time.

In UDC, serum specimens (and plasma specimens during 1998–2004 to preserve viral RNA) were centrifuged and shipped to CDC on cold packs within 30 hours of the blood draw. CDC tested specimens using a panel of tests for hepatitis A and B viruses, HCV, and HIV. Testing for HCV RNA represented a unique effort to detect presence of virus in a specimen between the time of exposure and antibody development, which enhanced surveillance for blood product safety in a population known to be vulnerable to this risk.

In Community Counts, serum specimens are similarly collected and submitted to CDC for HCV and HIV testing. In addition, plasma specimens are collected and submitted to CDC, where they are tested for the presence of inhibitors (antibodies) to hemophilia factor-replacement treatment products. Inhibitor development occurs in up to one third of persons using hemophilia treatment products, with the highest rates occurring in persons with the most severe cases of hemophilia (*17*). Although some inhibitors are transient, in a substantial proportion of cases, inhibitors have long-term clinical impacts, rendering treatment products ineffective at controlling bleeding. Plasma that has been separated from a centrifuged blood specimen is shipped on cold packs within 72 hours or is frozen and shipped on dry ice within 30 days of the blood draw.

The CDC surveillance laboratory has a Clinical Laboratory Improvement Amendments (CLIA) certification and conducts tests using FDA-approved kits or CLIA-approved in-house developed tests. Two-factor identification of specimens using the patient unique identifier and date of birth linked to an internal specimen number is used to meet CLIA requirements for reporting results.

Serum and plasma specimens that are not used in current viral and inhibitor tests are stored at CDC for future testing. The specimens in the repository are not linked to personally identifiable information; however, as with other registry data, HTCs are able to link the unique patient identification numbers to their patients’ personal identifiers. The CDC specimen repository is a resource to facilitate rapid investigation of new and emerging bloodborne agents and complications that might pose a serious potential risk for persons with bleeding disorders. Only markers and agents that are considered to constitute a credible public health risk for the hemophilia population are deemed to qualify for surveillance testing and reporting. Examples include previously unknown transmissible agents, biologic markers for chronic health conditions that might affect the aging hemophilia population, and markers related to new complications of hemophilia or hemophilia treatments. The specimens are stored indefinitely unless otherwise requested by participants.

### Surveillance Data Reports

Numerous reports from the UDC system have been published; these include the findings of specific UDC analyses and overviews of specific aspects of UDC. Examples of these publications are provided (Box 2) (https://www.cdc.gov/ncbddd/hemophilia/articles.html). Community Counts data reports also are periodically published on the CDC website. Recently, CDC launched a data visualization tool (https://www.cdc.gov/ncbddd/hemophilia/communitycounts/data-viz.html) to allow clinicians, patients, policymakers, and the general public to access Community Counts deidentified data within the same calendar year as submission to CDC. Users can tailor the data display using filters to specify subgroups and characteristics of interest including demographics, clinical diagnoses, treatments, and complications.

BOX 2 **Contributions of CDC hemophilia surveillance data to blood product safety, health of persons with hemophilia, and laboratory testing guidelines**

**U.S. blood safety**

- Data from the Universal Data Collection (UDC) system demonstrated that since 1998, no new infections of hepatitis A, B, or C, or human immunodeficiency virus (HIV) have been linked to blood products to treat hemophilia.
- Data from UDC demonstrated that parvovirus B19 transmission was occurring via plasma-based treatment products, even after viral inactivation processes were enacted to prevent the transmission of HIV and hepatitis.
  - Results of early UDC studies of parvovirus B19 transmission led to uniform screening of plasma pools for the B19 virus.
  - Subsequent analyses of UDC data have indicated that although the screening strategy likely led to a marked reduction in virus transmission, transmission was not completely eliminated.

**Joint health**

- UDC analyses include some of the most comprehensive assessments to date of how hemophilia affects joint health. Among other findings, the data have established the following:
  - Joint hemorrhages and consequent invasive orthopedic procedures are common in persons with moderate and severe hemophilia.
  - Impacts of joint bleeding in persons with hemophilia, as evidenced by joint range of motion limitations, begin at early ages, especially if factor deficiency is in the severe or moderate range.
  - High body mass index (in the overweight and obese range) is an independent risk factor for joint disease in persons with hemophilia. These data have important public health implications because persons with hemophilia might tend to have a sedentary lifestyle in an effort to prevent bleeding episodes associated with sports and other physical activity.
  - Septic arthritis is 15–40 times more common in the hemophilia population than the general population, with prevalence increasing as patients age.
  - Continuous prophylactic treatment, as opposed to episodic treatment (i.e., treatment of bleeding episodes when they occur), is associated with a substantial reduction in adverse joint health outcomes.
  - Despite the evidence in favor of prophylactic treatment, the treatment is used by a minority of persons with hemophilia, including 45% of persons with severe hemophilia B. This might be partly explained by health insurance and cost issues as the median annual cost for prophylaxis is 70% greater than the median annual cost for episodic treatment.
  - Nonetheless, a notable positive trajectory has occurred in access to standard of care therapy. For example, access to comprehensive care at young ages (i.e., ≤2 years) and use of a prophylactic treatment regimen were found to be more common in men born in 1976 or later than men from earlier birth cohorts.

**Hemophilia complications in infants and children**

- UDC analyses documented the unique complication risks that hemophilia poses for infants and children aged <2 years. Complications experienced in this age group were found to be distinct from those seen in older children and adults. For example, intracranial hemorrhage was common, occurring in approximately one third of infants studied.

**Development of inhibitors to hemophilia treatment products**

- A UDC analysis documented that patients with inhibitors (antibodies) to hemophilia treatment products had a 70% higher mortality rate than those without inhibitors.
- Data from UDC served as a sampling base for a more in-depth study, the Hemophilia Inhibitor Research Study (HIRS), of the development of inhibitors. Data from HIRS have been used to accomplish the following:
  - Study and refine laboratory techniques for detecting inhibitors.
  - Better understand which persons are at risk for developing inhibitors; for example, HIRS analyses demonstrate that persons with both mild and severe hemophilia are at risk.
  - Demonstrate relationships of gene variants to inhibitor risk in the United States.

**Empiric data that informed development of laboratory testing guidelines and of Community Counts**

- HIRS data informed guidelines issued by the Medical and Scientific Advisory Council (MASAC) of the National Hemophilia Foundation. Based on empiric review of data CDC provided from HIRS analyses, MASAC recommended annual inhibitor testing for persons with hemophilia using standard, state-of-the-art techniques. The laboratory assays were developed in part by CDC to ensure the accuracy and reproducibility of inhibitor tests among patients currently undergoing treatment.
- In addition, given the high, often prohibitive costs of setting up inhibitor testing at local laboratories and the lack of health insurance coverage for inhibitor tests, CDC added inhibitor testing as a component of Community Counts. Thus, the laboratory component of Community Counts provides both of the following:
  - Invaluable public health data to track a common, often debilitating hemophilia treatment complication.
  - Vital direct service to the hemophilia population.

## Evaluation of UDC and Community Counts

The UDC and Community Counts surveillance systems have several important attributes that have led to the long-term success of CDC’s hemophilia surveillance program. To assess performance of the CDC hemophilia surveillance systems, CDC conducted an evaluation in September 2019 using the CDC guidelines for evaluating public health surveillance systems (*18*). The attributes assessed were usefulness, simplicity, flexibility, data quality (completeness and validity), acceptability, sensitivity, predictive value positive, representativeness, timeliness, and stability. The evaluation included reviews of the system protocol, electronic data system, published research and surveillance reports, and data visualization tool. In addition, new analyses 1) assessed trends in key health indicators by combining data from UDC and Community Counts; 2) assessed the participation rates and representativeness of the current Community Counts patient registry; and 3) evaluated key indicators for system timeliness.

### Usefulness

A public health surveillance system is useful if it contributes to the prevention and control of adverse health-related events, including an improved understanding of the public health implications of such events (*18*). Data generated from UDC have been used in the development of many public health and clinical practices and guidelines. The large UDC sample size enabled key assessments related to U.S. blood product safety (*10*,*19*,*20*), hemophilia complications related to joint health (*5*,*21*–*28*), hemophilia complications unique to infants and children (*29*), and the development of inhibitors to hemophilia treatment products (*30*) (Box 2). Moreover, data from the Hemophilia Inhibitor Research Study (HIRS), for which UDC served as the sampling base, have allowed CDC to develop and validate key laboratory methods for inhibitor testing (*31*–*36*) and understand which patients were at risk for inhibitor development (*37*–*41*). This work was used for the development of key laboratory testing guidelines for the hemophilia population (*42*) and provided the empiric basis for expansion of laboratory testing in the development of Community Counts. The laboratory component of Community Counts provides essential public health data to track a common, often debilitating hemophilia treatment complication (development of inhibitors) and also provides a vital direct service to the hemophilia population. Because of the high, often prohibitive costs of setting up inhibitor testing at local laboratories and the lack of insurance coverage for inhibitor tests, inhibitor testing is an important benefit for the Community Counts participants.

**FIGURE 1:**
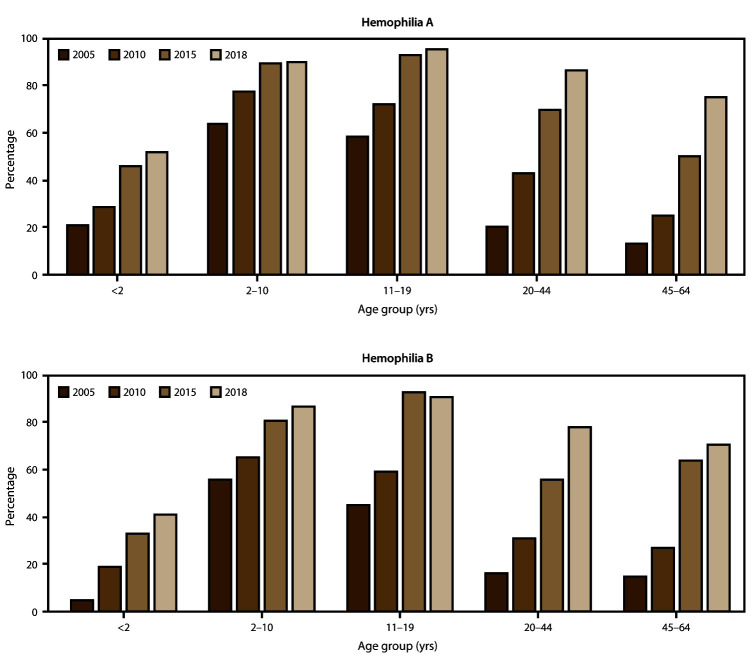
Percentage* of male registry participants with severe hemophilia who used a continuous prophylaxis† treatment regimen, by hemophilia type and patient age — Universal Data Collection surveillance system (2005 and 2010) and Community Counts surveillance system (2015 and 2018), United States * Data for this figure are available (Supplementary Table 1; https://stacks.cdc.gov/view/cdc/85148). ^†^ Patients were considered to be using continuous prophylaxis if the hemophilia treatment center provider indicated that the patient regularly used a treatment product to prevent bleeding episodes (even if the patient did not completely adhere to the regimen) and that the patient was expected continue with this regimen indefinitely.

**FIGURE 2:**
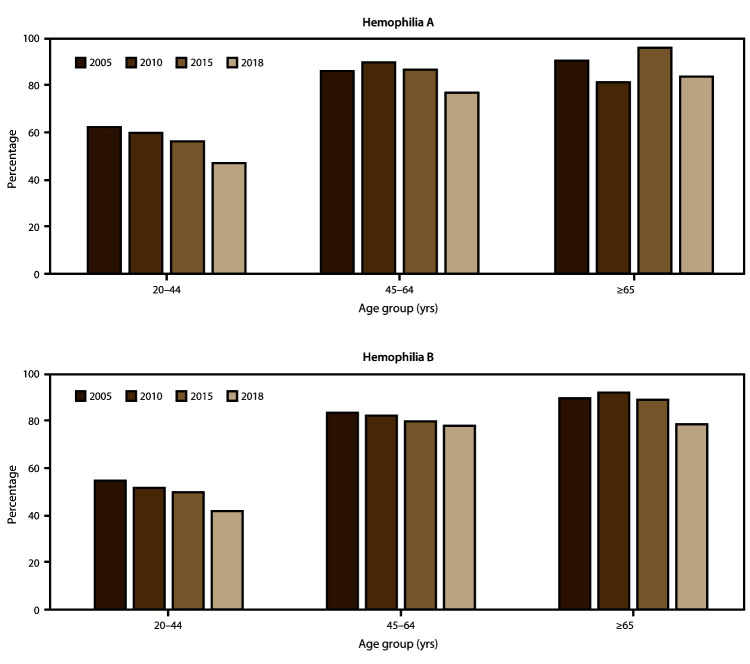
Percentage* of men aged ≥20 years with mobility limitations**†** among registry participants with severe hemophilia, by hemophilia type and patient age — Universal Data Collection surveillance system (2005 and 2010) and Community Counts surveillance system (2015 and 2018), United States * Data for this figure are available (Supplementary Table 2, https://stacks.cdc.gov/view/cdc/85148). ^†^ Mobility limitation in 2015 and 2018 was ascertained in Community Counts by responses to the following statement: “Check the statement which best describes how pain, loss of motion, or weakness due to joint disease affects the patient’s current overall level.” Response options included 1) unrestricted school or work and unrestricted recreational activities; 2) unrestricted school or work with limited recreational activity levels; 3) limited school or work and limited recreational activity levels; 4) limited school or work, limited recreational activity levels, and limited self-care activity levels; 5) requires assistance from another person for school or work or self-care, and unable to participate in recreation; and 6) unknown. Responses of unknown were excluded from the analysis. Participants who chose responses 2–5 were classified as having a mobility limitation in this analysis. Mobility limitation in 2005 and 2010 was ascertained from the Universal Data Collection (UDC) surveillance system using a very similar statement, with only minor verbiage differences between the UDC and Community Counts mobility limitation items.

The scope and longevity of CDC’s hemophilia surveillance program allows CDC to track trends in important health outcomes. One such outcome is use of continuous prophylaxis among males with severe hemophilia (the recommended treatment regimen for severe hemophilia) (Figure 1) (Supplementary Table 1; https://stacks.cdc.gov/view/cdc/85148). Patients were considered to be using continuous prophylaxis if the HTC provider indicated that the patient regularly used a treatment product to prevent bleeding episodes (even if the patient did not completely adhere to the regimen) and that the patient was expected to continue with this regimen indefinitely. Another such health outcome is mobility limitation in adulthood (Figure 2) (Supplementary Table 2; https://stacks.cdc.gov/view/cdc/85148). The combined data from the UDC and Community Counts systems allows for assessment of long-term trends. The large sample size allows for examination of trends within important patient subgroups, such as type of hemophilia and patient age. Recent trend analyses illustrate that among males with severe hemophilia, use of prophylactic treatment regimens has markedly increased in recent years across all age groups and for both hemophilia A and B (Figure 1) (Supplementary Table 1; https://stacks.cdc.gov/view/cdc/85148). This increase in prophylaxis use is temporally associated with positive health effects; the proportion of mobility limitations in men with severe hemophilia A and B have begun to decrease, most notably in young men (Figure 2) (Supplementary Table 2; https://stacks.cdc.gov/view/cdc/85148). Reported mobility limitations among men aged 20–44 years with severe hemophilia A and B decreased by 25% and 23%, respectively, from 2005 to 2018. Studies in progress using Community Counts data include examination of health outcomes among infants and young children with hemophilia, assessment of the epidemiology of inhibitor development in persons with severe hemophilia, and an examination of the efficacy of the current inhibitor testing protocol at reducing false-positive results.

### Simplicity

The simplicity of a public health surveillance system refers to both structure and ease of operation (*18*). To meet the surveillance objectives of tracking complications of hemophilia and hemophilia treatment products, the UDC and Community Counts systems were developed to track persons over time, which increases the complexity of the system. Nonetheless, when designing the systems, CDC and its partners were aware that they needed to be easy to use to be acceptable to providers and patients. Thus, the core data collection for UDC was designed to be relatively simple. Over the years, as new health issues arose, additional data collection was added to the core system as supplemental data collection modules or time-limited special studies to maintain the simplicity of the core system while allowing for new health issues to be explored in a timely manner.

In designing Community Counts, CDC faced additional challenges. Hemophilia is now a chronic condition with more treatment options and a much longer life expectancy than in the past; thus, tracking health complications has become more complex. As a result, CDC focused on developing data collection processes that minimized the time required of HTC providers. For example, the Community Counts electronic data submission system maintained by ATHN is continually updated in real time to provide a listing of FDA-approved treatment products. In addition, the electronic system allows for some data items to be prepopulated.

### Flexibility

A flexible public health system accommodates changes in information needs or operating conditions with little additional time, personnel, or allocated funds (*18*). The hemophilia surveillance program at CDC has been extremely flexible and therefore able to respond to emerging health priorities:

- In 2003, UDC was expanded to include infants and children aged <2 years, allowing for tracking of persons with hemophilia from birth and the ability to better understand the health needs of this young group.
- In 2005, a quality of life questionnaire was introduced as an optional supplement to UDC registry data collection.
- In 2008, more detailed data on treatment product inhibitors were collected via HIRS, a special study using the UDC sampling frame.
- In 2010, CDC and HTC investigators established normal reference values for joint range of motion (ROM) in a supplemental study of healthy persons (i.e., those without conditions that could potentially limit joint mobility) (*43*). These data were invaluable to extending the investigators’ work on joint ROM limitations in UDC participants to better understand early (occult) joint disease in this population (*23*).
- In 2011, UDC was replaced by Community Counts, the next iteration of the surveillance system, a transition that is perhaps the most important example of the flexibility of the surveillance system. This change ensured that the latest priority health issues were monitored, including prevalence and complications associated with inhibitors to various new and traditional hemophilia treatment products.

### Data Quality

Data quality reflects completeness and validity of the data recorded in the public health surveillance system (*18*). Much of the clinical data on hemophilia-associated complications in UDC and Community Counts are directly collected by care providers, primarily from data included in patients’ medical records. Data on treatment products currently in use and current health conditions are likely valid because these indices are based on health data documented in the medical record at time of the patient’s visit. Nonetheless, some clinical data are not as easily ascertained or validated during the visit (e.g., past bleeding episodes and other historical clinical data). These types of data might be subject to recall or social desirability bias, particularly for patients whose HTC visits are infrequent (>1 year apart). Both UDC and Community Counts incorporate systematic data quality checks, such as those to flag out-of-range data. In Community Counts, the CDC surveillance team also runs a series of cross-checks of related variables to detect potential erroneous data entry. HTCs are asked to review and confirm or correct questionable data. Data reporting is complete for most data items, especially key clinical indicators, such as joint bleeding. (In 2014–2017, only 1% of those with severe hemophilia and 4% of those with mild hemophilia were missing data on history of joint bleeding.)

Blood specimens are analyzed by CDC using state-of-the-art technology. Numerous advanced inhibitor testing procedures that are considered the gold standard were, in part, developed by CDC scientists. Testing for inhibitors is inherently complex because laboratory procedures must be incorporated to ensure that treatment products being used by the patient do not interfere with the ability of the laboratory test to detect an inhibitor. Using the most up-to-date and appropriate tests is important; the alternative would be to have patients discontinue treatment for a period (i.e., a washout period), which clearly is not optimal. The preanalytic heat treatment procedure to limit interference by infused or endogenous factor VIII in the inhibitor assay is one example of an important innovation developed by CDC (*36*). A commercial kit based on these CDC-developed methods was recently introduced internationally (https://precisionbiologic.com/news-events/news/factor-viii-inhibitor-kit-cleared-sale-us). This will improve standardization and decrease interlaboratory variability, both of which will improve data quality.

To address certain data needs, special studies have been conducted. Some of these studies entailed collecting new detailed data (e.g., the development of normal reference measures to assess joint ROM) (*43*). Such data are often among the highest quality data available on certain health topics that are particularly pertinent to the hemophilia population.

### Acceptability

Acceptability reflects the willingness of persons and organizations to participate in the surveillance system (*18*). Perhaps the most important factor in the long-term and ongoing success of both UDC and Community Counts has been the collaboration between CDC and key stakeholders within the bleeding disorders community in system design, development, and implementation. Tracking complications associated with hemophilia and hemophilia treatment products required that the surveillance system incorporate a longitudinal clinical component, a major challenge for a public health surveillance system. USHTCN and its HTC regional core centers have been integral partners throughout the development, implementation, and maintenance of both UDC and Community Counts, and ATHN is an additional core partner in the establishment and maintenance of Community Counts.

In addition, CDC works with several other partner organizations that provide valuable insights into how surveillance activities can best meet the public health needs of the hemophilia population; these include the National Hemophilia Foundation (https://www.hemophilia.org), a leading hemophilia advocacy organization with chapters in nearly every state; the Hemophilia Federation of America (https://www.hemophiliafed.org), a patient education, services, and advocacy organization with chapters across the United States; and the American Society of Hematology (https://www.hematology.org), a professional medical society. CDC also has developed strong partnerships with other federal agencies involved in hemophilia, including HRSA, the agency that funds HTCs to provide optimal care using a multidisciplinary team approach, as well as the national infrastructure of regional hemophilia networks; the National Heart Blood and Lung Institute at the National Institutes of Health, which provides support for clinical, translational, and implementation research in bleeding disorders; and FDA, which provides regulatory oversight of hemophilia treatment products and therapies.

Analyses of 2016–2018 Community Counts data indicate that 49% of persons with hemophilia A and 42% of persons with hemophilia B who receive care at U.S. HTCs participate in the Community Counts patient registry (Table 2). Participation rates are particularly high for those with severe hemophilia (i.e., <1% clotting factor activity in the circulating blood); participation rates were 59% and 57% for those with severe hemophilia A and B, respectively. One reason for the high participation rates among these groups might be the direct benefits Community Counts provides to participants. Because of the complexity and range of treatment products on the market, conducting the latest inhibitor tests is not cost-effective for HTC laboratories. Thus, in addition to CDC’s focus on tracking inhibitors to inform public health guidance on reducing hemophilia complications, Community Counts inhibitor testing results are a valuable benefit to participants, many of whom do not have an alternative testing source.

**TABLE 2:** Comparison of patients served by hemophilia treatment centers with subset of patients who also participated in the Community Counts patient registry — 2016–2018, United States

Hemophilia type and patient characteristics	HTC population profile	Patient registry	Registry participation (%)
	No. (%)*	No. (%)	
**Hemophilia A**			
**Total no.**	**15,859**	**7,811**	**49.3**
**Factor deficiency level^†^**			
Severe	7,597 (47.9)	4,489 (57.5)	59.1
Moderate	2,601 (16.4)	1,293 (16.6)	49.7
Mild	5,418 (34.2)	1,991 (25.5)	36.7
Unknown	243 (1.5)	38 (0.5)	15.6
**Sex**			
Female	1,574 (9.9)	309 (4.0)	19.6
Male	14,285 (90.1)	7,502 (96.0)	52.5
**Age group (yrs)**			
<2	461 (2.9)	204 (2.6)	44.3
2–10	3,072 (19.4)	1,645 (21.1)	53.5
11–19	3,553 (22.4)	1,999 (25.6)	56.3
20–44	5,901 (37.2)	2,699 (34.6)	45.7
45–64	2,056 (13.0)	945 (12.1)	46.0
≥65	816 (5.1)	319 (4.1)	39.1
**Race**			
Asian/American Indian or Alaska Native/Native Hawaiian or other Pacific Islander	897 (5.7)	356 (4.6)	39.7
Black	1,803 (11.4)	950 (12.2)	52.7
White	12,371 (78.0)	6,078 (77.8)	49.1
More than one race	198 (1.2)	114 (1.5)	57.6
Unknown	590 (3.7)	313 (4.0)	53.1
**Ethnicity**			
Hispanic	2,994 (18.9)	1,286 (16.5)	43.0
Non-Hispanic	12,672 (79.9)	6,450 (82.6)	50.9
Unknown	193 (1.2)	75 (1.0)	38.9
**Insurance status**			
Insured	15,338 (96.7)	7,604 (97.3)	49.6
Uninsured	362 (2.3)	159 (2.0)	43.9
Unknown	159 (1.0)	48 (0.6)	30.2
**Factor deficiency level,^†^ by age group (yrs)**			
Severe			
<2	227 (3.0)	138 (3.1)	60.8
2–10	1,549 (20.4)	1,007 (22.4)	65.0
11–19	1,756 (23.1)	1,172 (26.1)	66.7
20–44	3,191 (42.0)	1,707 (38.0)	53.5
45–64	729 (9.6)	396 (8.8)	54.3
≥65	145 (1.9)	69 (1.5)	47.6
Moderate			
<2	90 (3.5)	36 (2.8)	40.0
2–10	526 (20.2)	273 (21.1)	51.9
11–19	571 (22.0)	327 (25.3)	57.3
20–44	919 (35.3)	417 (32.3)	45.4
45–64	352 (13.5)	181 (14.0)	51.4
≥65	143 (5.5)	59 (4.6)	41.3
Mild			
<2	130 (2.4)	29 (1.5)	22.3
2–10	953 (17.6)	359 (18.0)	37.7
11–19	1,200 (22.1)	494 (24.8)	41.2
20–44	1,699 (31.4)	559 (28.1)	32.9
45–64	927 (17.1)	360 (18.1)	38.8
≥65	509 (9.4)	190 (9.5)	37.3
**Hemophilia B**			
**Total no.**	**4,948**	**2,095**	**42.3**
**Factor deficiency level^†^**			
Severe	1,340 (27.1)	763 (36.4)	56.9
Moderate	1,797 (36.3)	749 (35.8)	41.7
Mild	1,742 (35.2)	572 (27.3)	32.8
Unknown	69 (1.4)	11 (0.5)	15.9
**Sex**			
Female	652 (13.2)	122 (5.8)	18.7
Male	4,296 (86.8)	1,973 (94.2)	45.9
**Age group (yrs)**			
<2	145 (2.9)	52 (2.5)	35.9
2–10	969 (19.6)	404 (19.3)	41.7
11–19	1,041 (21.0)	489 (23.3)	47.0
20–44	1,659 (33.5)	674 (32.2)	40.6
45–64	782 (15.8)	337 (16.1)	43.1
≥65	352 (7.1)	139 (6.6)	39.5
**Race**			
Asian/American Indian or Alaska Native/Native Hawaiian or other Pacific Islander	173 (3.5)	83 (4.0)	48.0
Black	383 (7.7)	184 (8.8)	48.0
White	4,251 (85.9)	1,749 (83.5)	41.1
More than one race	18 (0.4)	10 (0.5)	55.6
Unknown	123 (2.5)	69 (3.3)	56.1
**Ethnicity**			
Hispanic	496 (10.0)	221 (10.5)	44.6
Non-Hispanic	4,390 (88.7)	1,849 (88.3)	42.1
Unknown	62 (1.3)	25 (1.2)	40.3
**Insurance status**			
Insured	4,204 (85.0)	1,914 (91.4)	45.5
Uninsured	664 (13.4)	164 (7.8)	24.7
Unknown	80 (1.6)	17 (0.8)	21.3
**Factor deficiency level,^†^ by age group (yrs)**			
Severe			
<2	50 (3.7)	24 (3.1)	48.0
2–10	278 (20.7)	179 (23.5)	64.4
11–19	253 (18.9)	170 (22.3)	67.2
20–44	519 (38.7)	268 (35.1)	51.6
45–64	190 (14.2)	95 (12.5)	50.0
≥65	50 (3.7)	27 (3.5)	54.0
Moderate			
<2	42 (2.3)	19 (2.5)	45.2
2–10	365 (20.3)	141 (18.8)	38.6
11–19	402 (22.4)	185 (24.7)	46.0
20–44	581 (32.3)	227 (30.3)	39.1
45–64	274 (15.2)	130 (17.4)	47.4
≥65	133 (7.4)	47 (6.3)	35.3
Mild			
<2	48 (2.8)	8 (1.4)	16.7
2–10	312 (17.9)	82 (14.3)	26.3
11–19	378 (21.7)	134 (23.4)	35.4
20–44	538 (30.9)	177 (30.9)	32.9
45–64	301 (17.3)	107 (18.7)	35.5
≥65	165 (9.5)	64 (11.2)	38.8

**Abbreviation:** HTC = hemophilia treatment center.

* Column percentages might not total 100% because of rounding.

^†^ Hemophilia severity is defined by level of clotting factor activity in circulating blood and comparison to an international standard. Severe hemophilia is defined as <1% activity, moderate hemophilia is defined as 1%–5% activity, and mild hemophilia is defined as >5% activity. For the vast majority of persons classified as mild, factor level activity is ≤50%. However, 8.4% of persons in the HTC population profile and 1.8% of persons in the registry who are classified as having mild hemophilia have factor activity levels >50%; these persons are nonetheless reported by their HTC as having a diagnosis of hemophilia.

### Sensitivity and Predictive Value Positive

Sensitivity refers to the proportion of cases of a disease (or other health-related event) detected by the surveillance system (*18*). Sensitivity also can refer to the ability to detect outbreaks, including the ability to monitor changes in the number of cases over time. Predictive value positive (PVP) is the proportion of reported cases that have the health-related event under surveillance (*18*). In UDC and Community Counts, hemophilia patients’ current treatment and clinical data, such as mobility limitations, and measured health indices, such as weights and heights to calculate body mass indices, are likely to have high PVP. Moreover, tracking trends in these indicators is likely to have reasonable sensitivity. However, some health indicators that must rely on retrospective patient recall, such as previous bleeding episodes for which a patient did not receive care from the HTC, family history, and chronic pain and opioid use, are more subject to underreporting and incorrect reporting. Thus, these data items might have lower sensitivity and PVP than current health indicators recorded in the medical record.

The changes in inhibitor laboratory testing methods developed by CDC have minimized both false-negative and false-positive results. As a result, the current method both facilitates CDC’s surveillance for inhibitors and allows testing of patients without a factor washout period. Performing an empirical assessment of the sensitivity and PVP for the inhibitor testing methods used is difficult because they are considered gold standard methods. However, ancillary data support specificity and PVP of the methods. CDC’s current inhibitor testing methods have indicated that 26% of newly detected inhibitors identified using other methods were false-positive results (*35*). CDC’s work in understanding the validity of inhibitor testing methods is ongoing. The effectiveness of testing needs to be continually evaluated in the context of other newer modified longer-acting treatment products, now or soon to be available. Community Counts data will be an important component of these evaluations and subsequent laboratory assay development.

### Representativeness

Representativeness is the extent to which the surveillance system accurately describes the occurrence of a health-related event over time and its distribution in the population (*18*). The addition of the total HTC population profile data collection to Community Counts has allowed CDC to better understand how participants in the surveillance registry might differ from those who receive care in federally funded HTCs but do not participate in the registry. Comparison of the population profile and patient registry on the distributions of demographic factors indicates that the hemophilia patient registry population is generally similar to the total population from which it was drawn (Table 2). However, one exception to this comparability is the coverage of females in the Community Counts registry; although 10% and 13% of persons with hemophilia A and hemophilia B who received services at HTCs in 2016–2018 were female, only 4% and 6%, respectively, of registry participants were female.

Despite the comparability of the Community Counts registry and HTC population profile, because both the UDC and Community Counts registry data are limited to persons who receive care in federally funded HTCs and neither system covers persons with hemophilia who do not use HTCs, the findings from both systems might still underestimate the prevalence of persons affected by hemophilia and incompletely characterize clinical outcomes. However, empiric assessments of select states have suggested that surveillance systems based on HTCs capture the vast majority of persons with hemophilia living in the United States (*3*,*4*). The most recent assessment of HTC coverage is based on persons who lived in Indiana during 2011–2013; 82% received care at a federally funded HTC, and 94% of those with the most severe cases of hemophilia received care in HTCs (*4*). Therefore, the findings from analyses of the registry are likely generalizable to the larger hemophilia population in the United States. In addition, Community Counts, together with its predecessor, the UDC system, is among the largest data collection systems of persons with hemophilia worldwide.

### Timeliness

Timeliness refers to the speed between steps of the surveillance system (*18*). A notable strength of Community Counts is that data are submitted continuously rather than in batches for annual submissions. In the last full year of data collection, 60% of the clinical forms collected from patients at initial and annual HTC visits were submitted to CDC within 15 days of the visit; 76% were submitted within 30 days, and 87% were submitted within 60 days (Table 3). Lag times between patient visit and submission of blood specimens to the CDC laboratory were even shorter; 71% were submitted within 15 days, 86% were submitted within 30 days, and 94% were submitted within 60 days. The CDC laboratory tested and provided results back to the HTCs within 30 days for >98% of the specimens.

**TABLE 3:** Timeliness of Community Counts surveillance procedures — 2018*^,†^

Timeliness indicator	No. (%)
**No. of days between visit date and submission of clinical data to CDC (via ATHN)**	
<15	3,859 (60.1)
15–29	1,031 (16.0)
30–59	712 (11.1)
≥60	824 (12.8)
**No. of days between submission to CDC and availability to public via data visualization platform**	Data visualization data set updated monthly
**No. of days between visit date and submission of laboratory specimen to CDC hemostasis laboratory**	
<15	5,924 (71.1)
15–29	1,219 (14.6)
30–59	706 (8.5)
≥60	485 (5.8)
**No. of days between receipt by CDC hemostasis laboratory and reporting of results back to HTC**	
<15	7,803 (93.6)
15–29	337 (4.0)
30–59	84 (1.0)
≥60	110 (1.3)

**Abbreviations:** ATHN = American Thrombosis and Hemostasis Network; HTC = hemophilia treatment center.

* Some percentages might not total 100% due to rounding.

^†^ The total number of hemophilia visits reported to the Community Counts patient registry in 2018 was 6,426. Of these, laboratory specimens were submitted for 5,357 persons. Because more than one specimen could be submitted per person, the total number of specimens submitted to and tested by the CDC laboratory was 8,334.

The transition from UDC to Community Counts led to a lag in data collection while various data processes were implemented. However, CDC is prepared to produce ongoing timely surveillance reports. Moreover, the recent release of a public data visualization tool allows for maximum use of the data in near real time.

### Stability

A stable system is reliable and available (*18*). UDC was stable for over a decade in terms of both provider and patient participation and CDC’s ability to process and analyze the data. CDC has now collected Community Counts HTC population profile data for 7 years and clinical registry data and specimens for 6 years and continues to work with partners to revise the system for improved ease of use and navigability.

## Future Directions

Community Counts will continue to track complications associated with hemophilia and hemophilia treatment products and to release the data in near real time via an electronic data visualization tool. The shortened time frame from data collection to analysis and release maximizes the ability to detect unanticipated consequences associated with many new types of hemophilia treatment products that have become available or will soon become available. Although these products offer great promise in treating and preventing bleeding episodes, long-term tracking in the population is needed to understand both the benefits and potential unanticipated complication risks associated with these products, including how those benefits and risks might vary in demographic and clinical population subsets. CDC will continue to play a key role in providing the data critical to understanding and maximizing the health and longevity of persons with hemophilia.

## Conclusion

CDC’s hemophilia surveillance program was originally designed and subsequently updated with consideration of the most pressing health indicators to be examined and tracked for persons with hemophilia. The findings from the system can be used in the prevention and treatment of medical complications associated with hemophilia and hemophilia treatment products. Data generated from UDC, CDC’s first comprehensive hemophilia surveillance system, were used in the development of many public health and clinical guidelines and practices linked to improved safety of U.S. blood products and prevention and early identification of hemophilia-related complications. Community Counts was designed to align with the UDC system, allowing for longitudinal assessments across the years covered by both systems. In addition, Community Counts is an important expansion of UDC for capturing data relevant to the hemophilia population as it ages. The innovations incorporated into the Community Counts surveillance system were based on empiric assessments of data from UDC as well as discussion with partners at the HTCs and the wider bleeding disorders community to understand the priority needs of the hemophilia population going forward.

UDC and Community Counts have several important attributes that have led to their long-term success, including the following:

- A stable data collection design that was developed and is continually reviewed in close partnership with HTC regional leaders and providers to ensure surveillance activities are focused on maximizing the scientific and clinical impact
- Flexibility to respond to emerging health priorities through periodic updates to data collection elements and, as needed, special studies
- High data quality for many clinical indicators and state-of-the-art laboratory testing methods for hemophilia treatment product inhibitors (developed and refined in part based on CDC research)
- Timely data and laboratory specimen submission, analysis, and reporting
- The largest and most representative sample of persons with hemophilia in the United States and one of the largest and most comprehensive data collection systems on hemophilia worldwide

Moreover, CDC’s hemophilia surveillance program has several unique aspects, including data collection directly from clinical entities, collection of data from the same persons over time to fully understand the complications of persons with hemophilia and the specific health care needs at different life stages, and a laboratory testing component, which in addition to providing data for public health monitoring, provides a direct clinical service to the participating hemophilia patients.

CDC’s hemophilia surveillance program offers valuable insights into other efforts to develop, implement, and maintain a surveillance system to track rare diseases and associated morbidities, mortality, and health care needs. Although hemophilia is a rare disorder that affects a small segment of the population, the resulting illnesses, complications, and deaths often are considerable. Similarly, many other rare disorders have substantial health and other effects and collectively have a widespread impact on public health. Epidemiologic data are lacking for many rare disorders, which have been estimated to affect approximately 30 million persons in the European Union and 25 million persons in the United States (estimates based on definitions for rare disorders as conditions affecting one or fewer of 2,000 persons in the European Union and one or fewer of 1,250 persons in the United States) (*44*,*45*). In addition, because numbers of health care providers with the appropriate specialized medical expertise to treat many rare disorders are limited, persons with rare disorders often have delays in diagnosis and treatment, resulting in additional complications and other sequelae associated with delayed treatment. CDC’s hemophilia surveillance program can serve as an example of how to conduct surveillance for a complex chronic disease by involving stakeholders, improving and building new infrastructure, expanding data collection (e.g., new diagnostic assays), providing testing guidance, establishing a registry with specimen collection, and integrating laboratory findings in clinical practice for individual patients.
